# Risks to the community pharmacists and pharmacy personnel during COVID-19 pandemic: perspectives from a low-income country

**DOI:** 10.1186/s40545-020-00250-2

**Published:** 2020-07-15

**Authors:** B. Dzingirai, C. M. J. Matyanga, T. Mudzviti, M. Siyawamwaya, D. Tagwireyi

**Affiliations:** 1 School of Pharmacy, College of Health Sciences, University of Zimbabwe, Harare, Zimbabwe; 2 Institute of Science in Healthy Aging & Healthcare (SHARE), University Medical Centre Groningen (UMCG), Groningen, The Netherlands; 3 Clinical Pharmacology, College of Health Sciences, University of Zimbabwe, Harare, Zimbabwe; 4 Newlands Clinic, Harare, Zimbabwe

**Keywords:** COVID-19, Community pharmacists, Pharmacy personnel, Low-income country, Personal protective equipment

## Abstract

Coronavirus disease 2019 (COVID-19) is an infectious disease that has become a global pandemic. COVID-19 is spreading in Africa, and Zimbabwe has not been spared. The cases in Zimbabwe are mainly from imported cases due to high volume of travellers from the COVID-19 hotspots. In Zimbabwe, local transmission is also anticipated due to inter- and intracity travelling. Frontline health workers are at risk of infection due to contact with infected people as they discharge their duties. In this setting, the risk to community pharmacists and pharmacy personnel is poorly understood and characterised. This paper looked at the risks of infection that are peculiar to community pharmacy personnel and suggested some recommendations to reduce the risk to COVID-19 infection.

## Background

Coronavirus disease 2019 (COVID-19) is an acute respiratory disease caused by the novel severe acute respiratory syndrome coronavirus 2 (SARS-CoV-2) [1]. The disease manifests as an asymptomatic infection (mild), which can progress to respiratory failure (severe) [2]. The main symptoms are fever, dry cough, muscle ache, fatigue, and shortness of breath [3–5]. The clinical presentation resembles viral pneumonia. Less common symptoms include sore throat, dizziness, headache, runny nose, diarrhoea, nausea/vomiting, and dizziness. The complications of COVID-19 which may result in death include ventilator-associated pneumonia, acute respiratory distress syndrome, acute respiratory injury, acute renal injury, and septic shock [5].

COVID-19 is a new disease, and scientists are still trying to understand how it spreads. There is evidence that it is transmitted from one person to another through close contact and through respiratory droplets generated from coughing or sneezing [6]. The droplets can also be inhaled into the lungs. Transmission from asymptomatic persons has also been reported [7, 8]. Risk factors for severe COVID-19 include close contact with infected individuals, residence in or travel to affected areas 14 days prior to symptom onset, people aged 65 years and above, and people with underlying medical conditions.

The pandemic started in December 2019 in Wuhan, Hubei Province, China [9, 10], and has spread to the rest of the world. As of 30 March 2020, 740,235 cases and 35,035 deaths had been recorded globally [11]. The updated figures, demographic, and epidemiological data can be obtained from the Worldometer webpage [11]. In many countries outside China, the initial cases were imported cases. Local and community transmission then resulted in the greater spread of the disease. As with many other African countries, Zimbabwe is at risk of imported cases due to citizens and tourists travelling from COVID-19 hotspots. Of great significance is that as of 29 March 2020, South Africa had 1280 confirmed cases [11, 12]. Zimbabwe and South Africa share a border with high volume of human traffic due to trade, tourist, and personal travel. As the number of COVID-19 cases increased in South Africa, the President issued a 3-week national lockdown on 23 March 2020. The situation in Zimbabwe also changed, with 7 confirmed cases and 1 death as of 30 March 2020 [11, 13]. The President of Zimbabwe declared a 21-day national lockdown effective 30 March 2020.

Healthcare workers are at risk of contracting COVID-19 during the outbreak because they are on the frontline of the fight. In China, a reported 3000 health workers were infected and more than 22 died [14]. Community pharmacists are the first point of contact with health-seeking patients on the community and therefore at risk of contracting COVID-19 during an outbreak. In Zimbabwe, community pharmacies and pharmacists contribute over 70% [15] of drug supply needs of the population and hence are on the frontline also of the COVID-19 fight. The risk to doctors, nurses, and laboratory personnel who are in direct contact with hospitalised COVID-19 patients is well anticipated, defined, and prepared for. Personal protective equipment (PPE) is provided for nurses, doctors, and laboratory personnel by the government and international organisation. For pharmacy personnel, it is unclear. In the UK, the government plans to provide COVID-19 protection packs to community pharmacists similar to what was provided to the general practitioners [16]. The protection packs include gloves, aprons, and fluid-repellent masks [16]. In a low-income country, risks to community pharmacists may be poorly understood. This paper aims to review and highlight the risks that are peculiar to community pharmacists in low-income countries and suggest some recommendations to mitigate the risks.

## Risks to the community pharmacists and pharmacy personnel

### Pharmacists on the frontline in the community-healthcare interface

Community pharmacies are on the frontline of the interface between the community and healthcare. This is because of physical proximity and easier access to the public. In Zimbabwe, pharmacist consultation is free; hence, the public can easily access the pharmacy for management of minor ailments. A greater proportion of COVID-19 patients present with mild symptoms mimicking a cold or flu and requiring no hospitalisation. With mild symptoms, many patients will consult the community pharmacist and pharmacy personnel first, and without a confirmed diagnosis of COVID-19, such consultations put the community pharmacist and personnel at risk of contracting the virus. To further aggravate the risk to the pharmacy personnel, the pharmacy is a regularly visited place by the public. Practising social distancing when serving patients is not easy in a pharmacy setting. Daily, a community pharmacist can interact with an estimated minimum of 500 people consulting on cosmetics, minor ailments, and filling in of prescriptions. The people that walk in and out of the pharmacy are already self-selected from the general population because of one or two ailments. Having such a high number of “sick” people interacting in one place adds to the risk of infection to the community pharmacist.

### Risks due to the pharmacy design

The setup of the community pharmacy may contribute to the risks of infection [17]. Limited physical space and presence of many bench tops, chairs, and shelves can be contributing to the risk of infection. The small spaces may make it difficult to implement the 1–2-m social distance within the pharmacy. In Zimbabwe, the dispensary space is the one that is regulated to a minimum of 10 m^2^. The other spaces such the waiting area and over the counter areas have no stipulated requirements. As a way to limit rentals, many community pharmacies have very small spaces to aid social distancing. Also, COVID-19 transmission risk within the community pharmacy may be increased due the working surfaces such as patient chairs, over the counter tops, and shelves. Respiratory droplets and saliva can be deposited on these surfaces as clients talk with pharmacy personnel.

### Risks when restocking

COVID-19 pandemic resulted in extra demand for pharmaceutical products and sundries. Mainly, there is excessive demand for masks, gloves, antibiotics, vitamins, and sanitisers. Coupled to demand of products directly used in the fight against COVID-19 is panic buying of chronic medications as patients prepare for lockdowns of uncertain length [18, 19]. The extra demand strained already weak supply chain systems of pharmaceuticals in low- to middle-income countries (LMICs). Pharmacists and pharmacy personnel resultantly have a burden to order for restocking. Extra ordering and restocking put the pharmacists and personnel at risk of contracting COVID-19 as they receive stock from suppliers. Such risk is further aggravated by the fact that the manufacturing industry in LMICs is weak hence the need to source products from several vendors at a time.

### Travelling to work during lockdown

Most LMICs imposed total lockdowns as a way to slow down COVID-19 infections, flatten infection curve, and reduce pressure on healthcare facilities. Pharmacies and pharmacist are classified as essential services and remained open during the lockdown [20]. This means that pharmacists and pharmacy personnel continued travelling to work using public transport. When travelling to and from work daily, pharmacists and pharmacy personnel are at risk of infection from COVID-19 as they get in contact other essential service providers such as policemen, nurses, and food producers.

### Risks when dispensing prescriptions

The dispensing system in Zimbabwe involves filling in paper-based prescriptions that come in the form of single sheets from the doctor’s pad or thirty-two-paged hospital books that are used several times. The prescriptions pass through the hands of the patients, doctors, and nurses as the patient is being managed. The other health professionals in the patient management may have minimum protection such as gloves, gowns, and head gear. When the prescription is presented to the pharmacist and other pharmacy personnel for dispensing, it is a potential source of pathogens, COVID-19 included, putting the community pharmacy personnel at risk. In many developed countries, prescriptions are electronic thus eliminating this source of transmission.

The pharmacist also faces the pressure to dispense off-label medications to patients. Various therapeutic drugs have been repurposed and suggested for use in the treatment of COVID-19 patients. The list includes chloroquine, hydroxychloroquine, remdesivir, lopinavir, azithromycin, corticosteroids, zinc, and vitamin C [21–24]. None of the agents has been studied long enough to generate enough evidence for use. However, information on the suggestions, data from small studies, and statements from politicians reach the public through the media resulting in excessive demand for unapproved therapies. Pharmacists in Harare, Zimbabwe, have been overwhelmed with patients who have been requesting for chloroquine for treatment of COVID-19. Chloroquine is classified as a prescription preparation; hence, patients cannot purchase it over the counter. The pharmacist is faced with a difficult decision because most of the patients cannot afford to pay the consultation fees to see a doctor, yet the drugs require a prescription.

### Provision of point of care tests

The community pharmacies provide blood glucose, cholesterol, blood pressure, malaria, HIV, and pregnancy tests. Provision of these services has been found to be effective and cost saving [25, 26]. Most of these tests involve blood draw, testing, reviewing the history of the readings, and patient counselling by the pharmacist. The blood draw and the prolonged time of contact increase the risk of contracting COVID-19 to the pharmacist. Coupled to that is the impossibility of maintaining the required social distance when providing these services in the community pharmacy setting.

### Lack of guidance

The National Health Services (NHS), UK, drafted a complete guide of how the community pharmacies and pharmacist handle potential COVID-19 cases [27]. The document clearly guides the pharmacist on how to identify, isolate the individual, notify the relevant public health officials, and decontaminate the consultation room. The International Pharmaceutical Federation (FIP) also drafted a COVID-19 information and interim guidance for pharmacists and the pharmacy workforce [28]. The document provides relevant information and guidelines on COVID-19 in a primary care context and in hospital settings, and for pharmacists working as clinical biologists. In South Africa, the South African Pharmacy Council issued a brief guide to pharmacists and pharmacy staff concerning hygiene, PPE, and infection control in the workplace [29]. One of the reasons for lack of guiding documents in LMICs is the lack of expertise and resources to produce such documents in short space of time. In Pakistan, the pharmacy regulatory bodies partnered with UK pharmacists to come up with a local guiding document to protect pharmacy personnel from COVID-19 [30]. In Zimbabwe, there is no guideline or standard operating procedure specific to the community pharmacies on COVID-19. The lack of guidance increases the risk of infection to the community pharmacists. The COVID-19 is novel, and there are a lot of myths and misconceptions that are misleading. A lack of knowledge has been attributed to fear of infection by health workers in Ebola outbreak [31]. The documents from other countries and international guidelines may not be applicable to a low-income setting where there are significant differences in pharmacy practice, demographics, funding levels, and different COVID-19 transmission dynamics. There is therefore a need for relevant country-specific guidelines on how to handle COVID-19 in the community pharmacy. The FIP guidelines can be used to tailor guidelines relevant to the Zimbabwean context.

Coupled to lack of guidance, community pharmacists are at risk of social media-driven misinformation on the transmission, clinical progression, and treatment of COVID-19. Community pharmacists also do provide health information to the community and require access to accurate data to be able to fulfil that role. The spread of misinformation on COVID-19 hinders the response of community pharmacists to the pandemic. Rapid dissemination of scientific, peer-reviewed, and verified information is critical in reducing public panic, spread of misinformation, and providing guidance to community pharmacists [32].

## Recommendations

Pharmacy practice in Zimbabwe is governed by the Pharmacist Council of Zimbabwe (PCZ), Health Professions Authority (HPA), and Medicines Control Authority of Zimbabwe (MCAZ). Responsive guidelines and regulations can help reduce the risk posed by COVID-19 to the pharmacists. Guidelines come as educative guides that give community pharmacists clear steps to take when handling potential cases of COVID-19. According to the World Health Organization (WHO), all health workers on the frontline of the COVID-19 fight need training on infection prevention and control. Community pharmacists should be trained on infection prevention and control (IPC). Most of the community pharmacies are owned by sole proprietors with very limited funds to be able to provide PPE. The government and the international funders such as the United Nations and WHO need to provide PPE to the community pharmacists during the COVID-19 outbreak. The PPE pack should include gloves, masks, and gowns/aprons.

Besides what the regulators and the government can do to reduce risk, community pharmacists themselves need to implement infection control measures learnt during professional pharmacy training such as ensuring running water and soap is always available in the pharmacy and dispensary area. All individuals entering the pharmacy should wash hands with soapy water or alcohol-based sanitiser. Measures should be put in place to ensure that a limited number of people are allowed in the pharmacy per given time. Within the pharmacy, a distance of 2 m between clients and between clients and staff should be maintained. Floors and counter tops should be wiped with sodium hypochlorite, hydrogen peroxide, quaternary ammonium, or alcohol-based disinfectant after every 30 min or after serving a customer. Innovative ways to prevent contact include placing transparent glass or plastic over the dispensing area with a provision of a very small opening. Contact may be minimised when dispensing by utilising prescription drop in baskets where patients can drop off their prescriptions and the pharmacist can in turn drop the medications. Mobile phones are very common in Zimbabwe and can be utilised to order prescription refills via short message service (SMS) or WhatsApp, and pharmacy personnel will deliver grouped orders. The personnel delivering the order will need full PPE including mask, gown, googles, and gloves. The risk and corresponding recommendations have been summarised in Table 1.

**Table 1:** Summary of the risks and recommendations

Risk	Category	Recommended mitigation measure(s)
Risk of infection from an infected patient walking into the pharmacy	High risk	i. Use full PPE* [33] for suspected cases as defined by WHO ii. Notify the Ministry of Health on provided contact details iii. Disinfect the pharmacy after the Ministry of Health taskforce has taken over
Risk of transmission when providing point of care tests	High risk	i. Sanitise hands with alcohol-based sanitiser before and after performing the test ii. Staff performing tasks that result in direct contact with patients should put on full PPE* [33] iii. All patients walking into the pharmacy to put one mask, cloth mask, or even a homemade mask
Risk from contact with patient personal items such as prescriptions, clinic book, and laboratory results slips	High risk	i. Use of drop boxes or mobile platform to order drugs ii. Use of latex gloves when handling items from patients iii. Use of alcohol-based sanitiser after dispensing [30, 34]
Risk due to high volume of people walking into the pharmacy	Moderate risk	i. Use of a mobile platform to order drugs from the pharmacy and deliver the drugs [20, 34] ii. Use of physical barriers on doorways and allowing a few into the pharmacy depending on size ii. Enforcing social distancing in and out of the pharmacy by placing tape on the floors marking areas patients should stand iii. Serving patients and customers from the dispensing window iv. Reduce interaction between pharmacy staff and clients by encouraging payments by mobile platforms v. All customers being served in the pharmacy to put on masks
Risk of infection when travelling to work	Moderate risk	i. Staff rotation where staff teams take turns to come to work ii. Provide masks for staff which should be put on when travelling to and from work
Risk of infection when receiving stock	Moderate risk	i. Designate days for receiving stock ii. Staff members responsible for receiving stock to wear mask and latex gloves iii. After receipt of new stock, staff to wash hands with soap or alcohol-based sanitiser
Risk due to small size of pharmacy and working surfaces	Moderate risk	i. Serving customers from the window ii. Staggering the customers that come to pharmacy by allocating time slots to regular customers iii. Regular cleaning of working surfaces with disinfectant
Risk due to lack of guidance	Low risk	i. Responsible pharmacy professional and regulatory bodies to issue timely guidance documents ii. Pharmacist to consult international or regional guidance documents such as WHO guidance
Risk of pressure to dispense off-label drugs	Low risk	i. Medicines Regulatory Authorities to issue evidence-based timely guidance on the dispensation of chloroquine and other drugs suggested for use in the management of COVID-19

*Full PPE includes a gown, gloves, face mask, and a face shield or goggles

## Conclusion

COVID-19 epidemic cases have been recorded and are on the increase in Zimbabwe. Community pharmacists are at risk of contracting the virus from the workplace. A number of ways to reduce the risk have been suggested in this paper. The authors hope that through this review, specific guidelines tailored for a low-income setting will be established to minimise any possibility of rapid transmission of disease through the community pharmacy.

## Abbreviations

COVID-19: Coronavirus disease 2019
FDA: Food and Drug Administration
FIP: International Pharmaceutical Federation
HIV: Human immunodeficiency virus
HPA: Health Professions Authority
IPC: Infection prevention and control
MCAZ: Medicines Control Authority of Zimbabwe
NHS: National Health Service
PCZ: Pharmacist Council of Zimbabwe
PPE: Personal protective equipment
WHO: World Health Organization
